# Mucoadhesive Rifampicin-Liposomes for the Treatment of Pulmonary Infection by *Mycobacterium abscessus*: Chitosan or ε-Poly-L-Lysine Decoration

**DOI:** 10.3390/biom13060924

**Published:** 2023-05-31

**Authors:** Jacopo Forte, Patrizia Nadia Hanieh, Noemi Poerio, Tommaso Olimpieri, Maria Grazia Ammendolia, Maurizio Fraziano, Maria Gioia Fabiano, Carlotta Marianecci, Maria Carafa, Federico Bordi, Simona Sennato, Federica Rinaldi

**Affiliations:** 1 Dipartimento di Chimica e Tecnologie del Farmaco, Sapienza Università di Roma, Piazzale Aldo Moro 5, 00185 Rome, Italy; jacopo.forte@uniroma1.it (J.F.); patrizianadia.hanieh@uniroma1.it (P.N.H.); mariagioia.fabiano@uniroma1.it (M.G.F.); carlotta.marianecci@uniroma1.it (C.M.); maria.carafa@uniroma1.it (M.C.); federica.rinaldi@uniroma1.it (F.R.); 2 Dipartimento di Biologia Università di Roma “Tor Vergata”, Via della Ricerca Scientifica, 00133 Rome, Italy; noemi.poerio@gmail.com (N.P.); tommaso.olimpieri.6@gmail.com (T.O.); fraziano@bio.uniroma2.it (M.F.); 3 Centro Nazionale Tecnologie Innovative in Sanità Pubblica, Istituto Superiore di Sanità, Viale Regina Elena, 299, 00161 Rome, Italy; maria.ammendolia@iss.it; 4 Istituto dei Sistemi Complessi (ISC)-CNR, sede “Sapienza” and Dipartimento di Fisica, Sapienza Università di Roma, 00185 Rome, Italy; federico.bordi@roma1.infn.it

**Keywords:** liposomes, Rifampicin, polymer decoration, Chitosan, ε-poly-L-lysine, *Mycobacterium abscessus*, mucoadhesion

## Abstract

*Mycobacterium abscessus* (Mabs) is a dangerous non-tubercular mycobacterium responsible for severe pulmonary infections in immunologically vulnerable patients, due to its wide resistance to many different antibiotics which make its therapeutic management extremely difficult. Drug nanocarriers as liposomes may represent a promising delivery strategy against pulmonary Mabs infection, due to the possibility to be aerosolically administrated and to tune their properties in order to increase nebulization resistance and retainment of encapsulated drug. In fact, liposome surface can be modified by decoration with mucoadhesive polymers to enhance its stability, mucus penetration and prolong its residence time in the lung. The aim of this work is to employ Chitosan or ε-poly-L-lysine decoration for improving the properties of a novel liposomes composed by hydrogenated phosphatidyl-choline from soybean (HSPC) and anionic 1,2-Dipalmitoyl-sn-glycero-3-phosphorylglycerol sodium salt (DPPG) able to entrap Rifampicin. A deep physicochemical characterization of polymer-decorated liposomes shows that both polymers improve mucoadhesion without affecting liposome features and Rifampicin entrapment efficiency. Therapeutic activity on Mabs-infected macrophages demonstrates an effective antibacterial effect of ε-poly-L-lysine liposomes with respect to chitosan-decorated ones. Altogether, these results suggest a possible use of ε-PLL liposomes to improve antibiotic delivery in the lung.

## 1. Introduction

*Mycobacterium abscessus* (Mabs) is one of the most common fast-growing species causing a serious chronic infection, named nontuberculous mycobacterial (NTM) pulmonary diseases, which are often present in patients with underlying lung conditions, such as bronchiectasis, cystic fibrosis or chronic obstructive pulmonary disease (COPD) [1]. Since NTM pulmonary diseases involve airways, intracellular and even bronchiole-epithelial infection in some cases, treatment remains challenging and outcomes are often poor. This is partly due to the ability of Mabs to evade host defenses and antimicrobial therapy through extracellular persistence in biofilms and sequestration into macrophages. Moreover, the presence of a thick, lipid-rich, hydrophobic cell wall of Mabs reduces the penetration of antibiotics into intracellular spaces thus causing antibiotic resistance.

The efficient drug delivery to the site of infection is the key aspect in the treatment of Mabs infection. Inhalation is the elective route for pulmonary delivery for its capability to conjugate drug administration to the infectious site and control of the drug penetration to the lung [2]. Inhaled liposomes can effectively reach the lung down to intracellular spaces and macrophages where the Mabs may reside [3]. Drug encapsulation in liposomes with chemical–physical properties engineered for inhalation can offer further advantages as the increase of the therapeutic index of the drug by offering protection from degradation and increasing the ability to reach the target sites while minimizing systemic exposure [4]. A well-performed liposome aerosolization is able to produce droplets with a proper size to increase the target localization and to maximize their efficacy at lower drug dose, preventing, at the same time, the systemic degradation of antibiotics and their pulmonary clearance. Many different investigations showed the increased efficacy of inhalation of liposome-encapsulated antibiotics like amikacin in Mabs treatment, compared with systemic administration [5]. Several liposomal formulations based on phosphatidylcholine and cholesterol have been shown to efficiently entrap the poorly soluble Rifampicin antibiotic and reach the lung by nebulization with an effective target to macrophages and good antibacterial activity [6,7].

It has been reported that the therapy outcome may be significantly increased by improving the nebulization properties of liposomes by modification of the lipid composition or concentration, or by using a polymer coating, to tune the bilayer stiffness, vesicle stability and resistance to stress [2,8]. Inhaled liposomes have to overcome or interact with the physiological barrier of mucus, which can be overproduced in mucus-related disorders such as cystic fibrosis and COPD. Retention of liposomes within mucus is ruled by the interactions established with mucus components, and in particular, with mucins. Mucins are long polymeric glycoproteins with a peptide backbone rich in carbohydrates chains and terminated by sialic acid [9]. Conventional liposomes are readily trapped by the airway mucus and rapidly cleared from the lung via mucus clearance mechanism so they cannot distribute throughout the lung airways nor long-reside in the lung and/or reach the airway epithelium. To tackle this challenge, several strategies to enhance particle penetration through the airway mucus are explored. The most often used approaches consider the modification of the physicochemical properties of drug-delivery vectors to promote their interaction with airway mucus and the modulation of barrier properties of the mucus itself [10,11]. Polymers are widely employed to improve the mucoadhesion of liposomes and other nanocarriers, to prolong and intensify their contact with adsorption sites, overcoming the challenge of a short retention time and increasing drug concentration gradient [12]. The “first generation” of mucoadhesive polymers include carbomers, chitosan (Chit), sodium alginate and the cellulose-derivatives hydrophilic macromolecules containing numerous hydrogen bond forming groups [13]. The natural polysaccharide Chit is probably the most well-known mucoadhesive agent [14]. The mucoadhesive properties of Chit were demonstrated by Lehr et al. in 1994 [15]. Thenceforth, a plethora of studies on Chit and its derivatives exploded and presented their many properties, from biodegradability, biocompatibility, reduced allergenic behavior, permeability augmentation, immunogenic, to antioxidant, antifungal and antibacterial effectiveness, as recently reviewed by Elkomy et al. [16]. Last, Chit is able to improve mechanical resistance to nebulization and several many other physicochemical and biological properties, thus inspiring researchers in the attempt to reach new heights in the delivery of drugs and nutraceuticals across different routes [16,17]. This justified the enormous use of chitosan as coating agent for liposomes and other nanoparticles.

Recently, the naturally occurring homo-polyamide ε-poly-L-lysine (ε-PLL) has attracted an ever-growing interest in the field of biomedical application thanks to its biodegradability and biocompatibility for humans and water-soluble properties [18] accompanied to a broad-spectrum of antimicrobial activity [19,20]. In ε-PLL the L-lysine residues are linked together by amide bonds between the carboxyl group of one residue and the ε-amino group, which is positively charged at neutral pH. Since the earlier studies, its polycation nature and its capability to electrostatically adsorb on bacteria cell surface has been considered crucial for the observed antimicrobial activity [21], justifying its use as food additive [22]. Electrostatic interaction of ε-PLL has been also explored for DNA compaction and gene delivery [23]. The chemical structure of ε-PLL prevents hydrophobic interactions with lipid bilayers, contrary to what observed in α-poly-lysine [24], thus encouraging the use of ε-PLL for stabilization of liposomes [25] and niosomes [26,27]. Recently, ε-PLL has also been used as a novel biological adhesive [28].

In this context, the use of Chit and ε-PLL was explored for improving the properties of a novel liposomal HSPC-DPPG formulation entrapping Rifampicin, in view of an effective in vivo pulmonary administration. As reported in a previous investigation [29], anionic HSPC-DPPG liposomes proved a high antibacterial activity towards Mabs, at a lower entrapped Rifampicin concentration, with respect to free drug. The present investigation aims to exploit polymer coating to obtain mucoadhesive vesicles able to reach the lungs but also to reduce the interaction and residence in the nose. For this ambitious goal, the concentration of the mucoadhesive polymer plays a key role. In fact, the polymer-decorated aerosolized liposomes could avoid nasal localization, reach the lung (to avoid and limit the nose to brain route) and interact with lung mucus—thus enhancing their residence time—when a slight polymer coating is present. In other words, the liposome surface is “decorated” by the minimal amount able to confer mucoadhesion only for long residence time in the target site, this is what occurs in the lungs bypassing the nose “barrier”. Moreover, in other investigations, it has been observed that the presence of the Chit coating is able to improve the retention of Rifampicin in DSPC-PG-Chol liposomes during nebulization, thus pointing out the significant effect of the polymer [30]. For this reason, in this work low concentrations of different polymers (Chit and ε-PLL) have been considered in order to obtain polymer-decorated liposomes with mucoadhesive properties which maintain similar physicochemical features of the undecorated ones. In particular, the site, extent and efficacy of nanocarriers deposition after inhalation is also influenced by the particle/droplet size (hydrodynamic diameter) [2]. A successful deposition into deep lung requires the particles to be small enough to avoid deposition by inertial impaction on upper airways and pass through the lower airways. On the basis of our previous investigation on the complexation between liposomes and polyelectrolytes [26,27], a low concentration of the polymer is able to slightly “decorate” the vesicle surface, rather than densely coat it, without affecting their dimension or ζ-potential [31]. 

The aim of this work is to evaluate the effect of the polymer decoration in the overall effectiveness of the liposomal nanocarrier, considering its basic chemical–physical properties, the eligible administration route and the antibacterial effect on in vitro Mabs infection. The effect of the polymer has been evaluated by a deep characterization of the hydrodynamic size, ζ-potential, morphology, vesicle bilayer properties, mucoadhesion and antibacterial efficiency. It is observed that both polymers are able to confer mucoadhesion without compromising the stability of the nanocarrier and its drug entrapment; this effect is connected to the favorable electrostatic interaction between the amino residues on polymer backbones and the negatively charged group of the mucin, thus prolonging the residence time in the pulmonary region and consequently enhancing the bioavailability of drugs [32]. Our results indicate ε-PLL–decorated liposomes are effectively internalized and are able to maintain an effective antibacterial action upon Mabs infection of macrophages. 

Up to now, scarce attention has been deserved to improve therapeutic management of Mabs by exploiting pulmonary drug administration based on mucoadhesive Chit-decorated liposomes, as already carried out for tubercular bacteria by employing Chit coating of anionic liposomal formulations, or, more generally, nanocarriers [16,30]. On the other hand, ε-PLL as coating agent has been scarcely or not at all explored and this represents the novelty of the proposed investigation, whose future developments consider deepening the effect of size and charge of ε-PLL-decorated liposomes complexes on internalization in promonocytic THP-1 leukemia cell, as observed by some of us in past studies on monocyte-macrophage cells [27]. Our findings open the way for future investigations on this scarcely explored natural polymer and its potentiality, as, to the best of our knowledge, ε-PLL has never been used for improving mucoadhesion or other technologically relevant properties of liposomal formulations.

## 2. Materials and Methods

### 2.1. Materials

1,2-Dipalmitoyl-sn-glycero-3-phosphorylglycerol sodium salt-DPPG (molecular weight Mw = 745 g/mol) and hydrogenated phosphatidylcholine from soybean-HSPC (molecular weight Mw = 790 g/mol) were a gift from LIPOID GmbH (Lipoid GmbH, Frigenstr, Ludwigshafen, Germany). Rifampicin (RIF, nominal purity >97%), diphenylhexatriene (DPH), calcein, mucin and Hepes salt [*N*-(2-hydroxyethyl) piperazine-*N*′-(2-ethanesulfonic acid)] were purchased by Merck Life Science S.r.l (Milano, IT). Low molecular weight chitosan (Chit) was purchased by Sigma-Aldrich. The Chit solution was prepared by dissolving Chit in acetate buffer (0.2 M, pH 4.4) up to a final concentration of 3 mg/mL. The obtained solution was stirred overnight. ε-Poly-L-lysine (ε-PLL) was a gift from Chisso Corporation (Yokohama, JP). This polymer is produced by a mutant of *Streptomyces albulus* NBRC14147 strain [33]. It is composed of 25 to 35 L-lysine residues (Mw ≈ 4000) and the ε-amino group is positively charged at neutral pH. ε-PLL was in the basic form and it was converted to Cl salt by titration with HCl followed by extensive dialysis to eliminate the H^+^ excess.

### 2.2. Preparation of Liposomes and Polymer-Decorated Liposomes

Liposomes and RIF-loaded liposomes were prepared by Thin Layer Evaporation technique by considering equimolar mixtures of HSPC and DPPG, as reported in detail in our previous work [29]. Lipids were dissolved in a chloroform/methanol mixture (3:1 *v*/*v*) and 1-h evaporation of organic solvents under vacuum at 60 °C was carried out by a Rotavapor^®^ R-210 (Büchi-Italia S.r.l., Assago, Milan, Italy), followed by overnight removal at room temperature in a T51 glass oven dryer (Büchi-Italia S.r.l., Assago, Milan, Italy). For drug-loaded liposomes, 5 mg of RIF have been dissolved with lipids. Lipid film was hydrated in 5 mL Hepes buffer (pH = 7.4 0.01 M) by using a vortex mixer and a water bath (60 °C for 3 min). In order to limit lipids chemical degradation such as hydrolysis and/or oxidation, and to degas the hydrating buffer solution, the samples were sonicated by tip (amplitude 16%, temperature 4 °C, time minutes, pulse on 0.8 and pulse off 0.6) under continuous nitrogen flow.

Composition of liposomes suspension in Hepes buffer (pH = 7.4 0.01 M) is reported in Table 1. Unentrapped RIF has been removed by centrifugation at 18,000 rpm and 4 °C for 30 min (MPW-260R). 

For calcein RIF-loaded liposomes, liposomes have been hydrated by calcein solution (10^−2^ M). At this concentration the dye is self-quenched, and no fluorescence is observed. The unentrapped dye has been removed by extensive dialysis against Hepes buffer for 10 h using a dialysis bag with molecular weight cut-off (MWCO) of 1000. After preparation, all samples were stored at 4 °C until their use in different experiments.

Polymer coating of LipoRIF was obtained by adding a polymer solution prepared at a proper concentration, to the liposome suspension (prepared as described above), by considering mixing of equal volumes.

### 2.3. Determination of Drug Entrapment and In Vitro Drug Release

UV–Vis spectroscopy has been employed to evaluate the amount of RIF entrapped in liposomes, its stability on time upon storage at 4 °C or 25 °C and its release by in vitro experiments. The same protocol reported in our past study has been employed [29]. Determination of drug content and drug release in RIF-loaded liposomes and polymer-decorated RIF-liposomes have been carried out at different times after preparation (1, 30, 60, and 90 days). The absorbance of RIF at λ = 465 nm has been measured by diluting purified liposomes in Ethanol:Hepes 1:1 (vol:vol), with Ethanol:Hepes 1:1 as reference. 

In vitro drug release was investigated by a dialysis test carried out at 37 °C in Hepes buffer (10 mM, pH 7.4). The dialysis bag was placed in a medium consisting of the Ethanol:Hepes 1:1 mixture and kept in continuous magnetic stirring for a time interval of 48 h. A cellulose acetate membrane with cut-off 8000 MWCO and diffusing area 5.5 cm^2^ has been used. To determine the amount of RIF released, aliquots of 1 mL were withdrawn from the release medium to perform UV analysis as described above, and then re-inserted back in the external medium. Analysis has been performed immediately after sampling, at different time points. 

*E.E.* was calculated as:

(1) $$E.E.\;\left(\%\right)=\frac{Entrapped\;drug\;\left(mg\right)}{Total\;drug\;used\;\left(mg\right)}\times 100$$

Results are shown as the average of three different preparations ± standard deviation. 

### 2.4. Size and ζ-Potential Measurements 

A Malvern NanoZetaSizer apparatus (Malvern Instruments, Worcestershire, United Kingdom), 90-degree configuration equipped with a 5 mW HeNe laser (λ = 632.8 nm), has been used to measure the size and electrophoretic mobility of samples. Cumulant method has been used to get the values of the hydrodynamic diameter (D_H_) and the polydispersity index (PDI) [34]. For the determination of ζ-potential, electrophoretic mobility µ was converted into ζ-potential by the Smoluchowski relation ζ = µ η/ɛ, where η and ɛ are the viscosity and the permittivity of the solvent phase, respectively [35].

To determine the stability of the suspensions, size and ζ-potential of empty liposomes, RIF-loaded liposomes and Chit or ε-PLL decorated liposomes have been measured at different times after preparation (1, 30, 60 and 90 days), for samples stored at 4 °C or room temperature. It must be pointed out that storage at 4 °C is considered the best condition to limit lipid degradation phenomena such as oxidation and hydrolysis [36]. Size distribution and ζ-potential were also determined before and after nebulization by a jet nebulizer (Nebula Air Liquide Medical Systems S.p.A., Bovezzo, Italy), in order to evaluate the stability of decorated-liposomes. The sample was opportunely diluted in the same buffer used for its preparation. Size and ζ-potential measurements have been performed on three different sample preparations, at least. Results are shown as the average value ± standard deviation.

### 2.5. Bilayer Characterization by DPH Fluorescence Anisotropy

DPH fluorescence anisotropy has been used to characterize the bilayer of liposomes and RIF-liposomes and the effect of polymer coating. DPH-loaded liposomes were prepared by co-dissolution in the mixture of organic solvents of lipids and probe (2 × 10^−4^ M), as described previously [29]. 

The fluorescence anisotropy (*A*) was determined by the following ratio:

(2) $$A=\frac{\left(I_{VV\;}-I_{VH\;}\right)\times G}{(I_{VV}+\;2I_{VH})\times G}$$

where *I_VV_*, *I_VH_*, *I_HV_* and *I_HH_* are the intensities (λ_exc_ = 350 nm, λ_em_ = 428 nm) of the fluorescence measured by a LS5013 PerkinElmer spectrophotometer [29], with *V* (vertical) and *H* (horizontal) orientation of the polarized light. *G* = *I_HV_*/*I_HH_* factor is the ratio of sensitivity of the detection system. Results are shown as the average of three different preparations ± standard deviation.

### 2.6. Preparation of Mucin Solution and Mucoadhesive Studies

Mucin powder was dissolved in Hepes buffer to obtain a solution at 2 mg/mL, pH 6.0 and stirred overnight at 34 °C. Specific parameters, including temperature (30 °C), concentration of mucin (2 mg/mL) and pH value (6.3–6.7), had been controlled in the mucoadhesive study to mimic the conditions in the lung site. Mucin solution (2 mg/mL) was mixed with liposome and polymer decorated-liposome suspensions (1:1 volume ratio), respectively, and incubated at 30 °C [37]. Particle size and ζ-potential were measured at 0, 5, 10 and 15 min, to determine the time needed for liposome–mucin complex formation and the stability of the complex. In order to obtain information about the mucoadhesive capabilities of the samples, the interaction between LipoRIF and polymer-decorated LipoRIF and mucin were also evaluated by performing fluorescence turbidity measurements using luminescence spectrometer (LS5013, PerkinElmer, Waltham, MA, USA) at Ex/Em 600/600 nm [37]. The absorbance (A) of the complex formed by liposomes and polymer-decorated liposomes in the presence of mucin was measured at λ = 500 nm. Each value was compared with a “ideal” absorbance (A_ideal_) calculated by adding the individual measured absorbance values of mucin and of each sample (LipoRIF, LipoRIF + Chit and LipoRIF + ε-PLL). The difference in absorption (ΔA = A − A_ideal_) was taken as a measure of the interaction between mucin and Lipo RIF, LipoRIF + Chit and LipoRIF + ε-PLL, namely ΔA ≅ 0 if no interaction occurs, while if ΔA > 0, a strong interaction between mucin and the analyzed samples was inferred. Results were obtained as the average of three independent experiments and values presented as the mean ± standard deviation.

### 2.7. Morphological Investigation 

Transmission Electron Microscopy (TEM) and Atomic Force Microscopy (AFM) were used to visualize the morphology of the liposomes as well as their interaction with culture medium. Samples for TEM were prepared on Formvar/carbon-coated copper grids. An amount of 40 µL of liposomal suspensions was placed on a strip of parafilm and the grids were allowed to swim on the surface of the droplets for 5 min. After adsorption, the grids were sequentially moved onto a series of Hepes buffer droplets for washing, then the samples were stained with 2% phosphotungstic acid (PTA) solution adjusted to pH 7.2. After drying, samples were visualized by a FEI 280S Transmission Electron Microscope (FEI Company, Hillsboro, OR, USA) operating at 100 kV. For AFM measurements, liposomal suspension was diluted 50× with Hepes buffer and then incubated on freshly cleaved mica for 10 min. After liposomes adsorption, the substrate surface was washed three times with Milli Q-water to remove the non-adsorbed particles. AFM imaging has been performed in air and at room conditions. Images were analyzed using Gwyddion free software. Height Sensor images were processed by flattening and background subtraction; on some selected image portions, Prewitt horizontal filter was used for a better visualization of surface structure and enhance image contrast. Measurements were performed in Tapping Mode with a Dimension Icon (Bruker AXS, Billerica, MA, USA) instrument under ambient conditions, by using RTESP-300 (Brucker) probes characterized by a sharp silicon tip (nominal radius of curvature 10 nm).

### 2.8. Biological Evaluation

#### 2.8.1. Bacterial Strains

*Mycobacterium abscessus* by American Type Culture Collection ATCC19977. Bacteria were stored and grown as previously described [38].

#### 2.8.2. Cell Line

Human promonocytic THP-1 leukemia cell line was supplied by the European Collection of Cell Culture, and were cultured as in [39]. In particular, for the experiments, cells were seeded in 24-well plates at the concentration of 5 × 10^5^/mL or in 96-well plates at the concentration of 2 × 10^5^/200 µL for 72 h in the presence of 20 ng/mL Phorbol 12-Myristate 13-Acetate (Merck Life Science S.r.l, Milano, Italy), getting differentiated THP-1 (dTHP-1). 

#### 2.8.3. Infection with Mabs 

dTHP-1 cells (5 × 10^5^/mL) were infected for 3 h with Mabs at the multiplicity of infection (MOI) of 10 at 37 °C with 5% CO_2_, in absence of antibiotics. Afterwards, extracellular mycobacteria were killed by 1 h incubation with 250 µg/mL Amikacin (Merck Life Science S.r.l, Milano, Italy) and cells were treated for 18 h with empty liposomes, lipo-RIF, polymer-coated lipo-RIF or free RIF. Rifampicin, where present, was used at 96 µM, which is the most effective concentration on the basis of our previous investigation [29]. Finally, intracellular bacterial growth was assessed by Colony-forming unit (CFU) assay; cells were lysed with 1% deoxycholate (Merck Life Science S.r.l, Milano, Italy), samples diluted in PBS–Tween 80 (0.01%) and CFU quantified by plating bacilli in triplicate on 7H10 supplemented with OADC.

#### 2.8.4. Stability of Liposomes in Culture Medium

As preliminary biological evaluation, the in vitro stability of liposomes in the presence of THP-1 culture medium has been carried out. LipoRIF, as well as empty liposomes, were diluted in culture medium to obtain a final concentration of 45%. The average size, polydispersity index, and ζ-potential were evaluated by means of DLS maintaining samples at 37 °C and performing measurements at different time points (8, 24, 28, 72 h).

#### 2.8.5. Uptake of Liposomes in dTHP-1 Cells 

dTHP-1 (5 × 10^5^/mL) were stimulated for 18 h with empty liposomes, LipoRIF or polymer coated LipoRIF, containing or not calcein. The internalization was analyzed by a flow cytometer FACS Celesta (Becton Dickinson, Franklin Lakes, NJ, USA) by considering the mean fluorescence intensity (MFI) of calcein-positive cells. Rifampicin concentration was fixed at 96 µM.

#### 2.8.6. Statistical Analysis

Results of biological characterization are expressed as the mean of two or three inde-pendent experiments ± standard deviation. The statistical analysis of biological data was performed by using two-tailed Student’s *t*-test.

## 3. Results and Discussion

### 3.1. Physicochemical Characterization of Liposomes

In this investigation, the concentration of Chit and ε-PLL polymers has been adjusted in order to introduce a minimal modification to the HSPC-DPPG liposomal formulation entrapping Rifampicin (RIF), which showed effective antibacterial activity [29] and obtain polyion-coated liposomes with sub-micrometric size and long-time stability [26,27,40]. Furthermore, a preliminary evaluation showed that high concentrations of Chit [1.35 mg/mL] or ε-PLL [0.9 mg/mL] may exert a cytotoxic effect on dTHP-1 cells. Table 2 reports the values of hydrodynamic diameter (D_H_), polydispersity index (PDI), ζ-potential, RIF entrapment efficiency (EE%) and anisotropy of Chit/ε-PLL coated liposomal formulation (see Appendix A for graphs of D_H,_ PDI and ζ-potential). Results of analysis of DLS size distribution are reported in Supplementary Materials.

It is immediately observed that the addition of polymers increases the hydrodynamic size with respect to the uncoated sample, in a different extent depending on the used polymer. Additionally, the ζ-potential increases with respect to the one of the uncoated sample LipoRIF (−45 mV), consequently to polyion adsorption. The observed variation of ζ-potential is small since the used polyion concentration is very low and liposome surface is only slightly modified with a polymer decoration, rather than densely coated as expected at higher polymer concentration, where saturation and overcharging occurs as described by Zaru et al. [30]. As it was in our intention, the obtained ζ-potential values for Lipo-RIF + Chit/ε-PLL were sufficiently negative to assure their stability [26] and avoid formation of clusters and destabilization [30]. The invariance of size and ζ-potential suggests that degradation due to lipid oxidation or hydrolysis are absent, as expected on the basis of preparation protocol on nitrogen flux and storage at 4 °C. Note that the PDI index remained indicative of a relatively narrow distribution (see Supplementary Materials for analysis of size distribution) which is a relevant aspect for drug pharmacokinetics. It is recognized how the suitability of nanocarrier formulations for a particular route of drug administration depends on their average diameter, but also by PDI and stability [41]. The control of these parameters is a key factor for the clinical efficacy and to predict or understand in vivo behavior of nanocarrier formulations. 

Chit/ε-PLL do not influence the EE% of Rifampicin or the anisotropy of the decorated liposomes, whose values are indicative of a rather rigid and ordered bilayer [42]. Actually, the observed slight increase of anisotropy induced by Chit can be due connected efficient adsorption of the polymer chains, favored by the low concentration and the linear conformation of chains, as reported by Tan et al. for anionic liposomes composed by PC and phosphatidic acid [43]. In this regime, it was observed that liposome morphology is unchanged and bilayer becomes more rigid. At increasing concentration, polymer conformation changes and may affect the fluidity in opposite way, with reduction of entrapment efficiency. 

All these findings support the surface character of the polymer decoration and indicate that its presence has no detectable influence on the intimate structure of the liposomal bilayer. Last, the measured pH value of the formulations (Table 1) confirms that both LipoRif + Chit/ε-PLL are suitable for nasal administration where a pH in the range of 3.5–6.4 is required [44].

In view of a possible administration by pulmonary route, the samples were nebulized to test the compatibility with this administration method. The evaluation of all the physical–chemical features of the formulations has been repeated after nebulization (Table A1 Appendix A). This investigation is relevant since it is well known that liposomes may be physically unstable during nebulization as a consequence of the shearing within the nebulizers which can disrupt lipid bilayers and fragment the vesicles, thus causing a marked loss of the entrapped hydrophilic molecules [45,46,47]. The investigated formulations do not undergo any relevant variation of size and ζ-potential (Table A1 Appendix A). This suggests that polyion decoration does not undergo peeling from the liposome surface. Furthermore, polyion-decorated liposomes do not undergo coalescence phenomena during nebulization and the small amount of polyion added to confer mucoadhesion does not vary the stability of polymer-coated liposomes after nebulization. Last, the amount of entrapped drug is unchanged and remains very high. 

All these findings indicate that both uncoated and polyion-decorated HSPC:DPPG liposomal formulations are very resistant to nebulization stress due to their high bilayer rigidity and charge. This is not surprising since it was reported from the earlier investigation that lipid composition, charge and bilayer packing have a relevant effect on stability during nebulization and drug entrapment properties [46]. Interestingly, for PC-based liposomal formulation entrapping RIF, it has been found that rigid liposomal membranes as those composed of distearoyl-glycero phosphocholine (DSPC), which is the 95% of lipid present in HSPC, showed an increased drug retention compared to phosphatidylcholine liposomes [48]. Furthermore, in mixed HSPC-Cholesterol-Oleic acid liposomal formulations entrapping Rifampicin, an enhanced resistance to nebulization has been observed for those mixtures of lipids with higher bilayer packing [49]. The role of bilayer rigidity has been pointed out in a comparative study between conventional SPC:Cholesterol liposomes and ultradeformable liposomes formulated with the addition of Tween 80 surfactant [47], whose results clearly showed the superior performance of rigid liposomes with respect to ultradeformable ones, which were less resistant and aggregate during nebulization. On these bases, it is possible to conclude that Chit and ε-PLL decorated liposomes are suitable for nebulization in order to potentially enhance the local drug availability to the infectious site in the lung.

### 3.2. Mucoadhesion Study

The investigation on the mucoadhesive properties was carried out to obtain information on the interaction between decorated liposomes and mucin. The ability to interact with mucin could represent a key feature for in vivo efficacy of the nanocarriers designed for lung delivery. For this purpose, the hydrodynamic diameter, ζ-potential, pH and turbidity values have been measured after mucin addition to polymer-decorated liposomes and the obtained data are reported in Table 3 and in Figure A2 (Appendix A). After mucin addition, the size increases significantly for all the samples – and furthermore the PDI also increases independently of the presence and nature of polymer. ζ-potential slightly decreased as a consequence of liposomes–mucin interaction since the presence of the cationic polymers promotes the adsorption of mucin on liposomes.

Moreover, to evaluate and compare the mucoadhesion property of decorated liposomes with respect to bare ones, the vesicles are prepared adding mucin to the samples, as described in the Materials and Methods section, and ΔA values are collected. Results have been reported in Table 3. It is possible to observe that only for the decorated vesicles the ΔA values are >0 [37,50], thus confirming liposomes–mucin interaction, while the uncoated liposomes are characterized by ΔA ≅ 0. The percent variation, ∆A % = ∆A/A_ideal_ × 100, was also evaluated. These results suggest that LipoRIF + Chit/ε-PLL are characterized by better mucoadhesion performances in the investigated experimental conditions. According to the literature, the adsorption of mucin on charged nanocarriers is affected by electrostatic interaction between mucin and nanocarrier surface [32]. Unfavorable electrostatic interaction between anionic Lipo-RIF and mucin reduces its adsorption. Conversely, since Chit (pKa ≈ 6) possesses strong positive charges due to the large amount of amine groups on the backbone, as well as ε-PLL, enhanced electrostatic interaction between mucin and these polymers results in the observed higher cohesive properties.

### 3.3. Morphological Investigation

Morphology of Chit/ε-PLL decorated liposomes have been studied by TEM and AFM, considering that the two techniques are able to give a complementary view of the polymer–vesicle complexes. 

**Figure 1:**
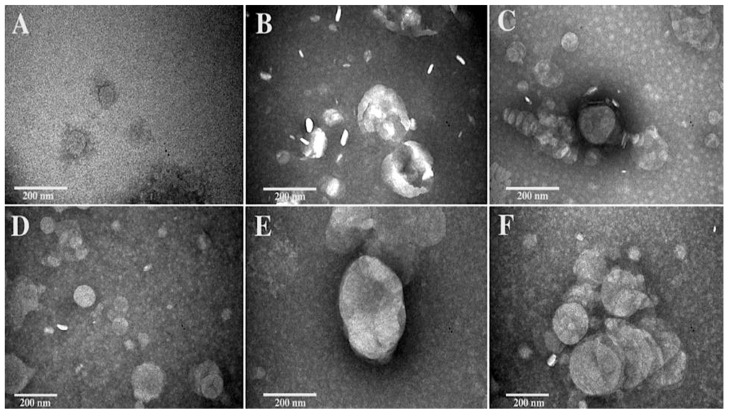
TEM images (PTA staining) of liposomal formulations, LipoRIF (**A**), LipoRIF-Chito (**B**), LipoRIF-ε-PLL (**C**) observed as prepared and after interaction with Mucin (**D**,**E**,**F**, respectively).

TEM images of LipoRIF and Chit/ε-PLL liposomes are shown in Figure 1, where for all the three samples, the upper panels refer to the suspensions as prepared and the lower panels reports their morphology observed after interaction with mucin. Figure 1, panel A shows the sample LipoRIF, in the image liposomes with spherical shape and an intact structure are observed. Although the image contrast is not so favorable, it is possible to observe the typical transparent structure of liposomes. The smaller size with respect to DLS hydrodynamic radius is due to dehydration and to intrinsic differences in size determination by the two techniques [51]. 

Interaction of RIF-loaded liposomes with Chit (Figure 1, panel B) results in an increased heterogeneity of the sample, as observed in TEM images. Interpreting this observation is a difficult task, since it is not possible to ascertain if the different objects coexist in bulk or form upon drying on TEM grid. In fact, it is well known that in suspensions of liposomes observed by electron microscopy, aggregation and deformation of vesicles are often observed as artifacts due to the staining process, as a consequence of the interaction between the sample and the staining molecule, and/or to the distortion and alteration caused by the drying steps which occur following the exposure of the sample to the vacuum [52]. DLS measurements confirm a general increase of size after polymer addition, with PDI values lower than 0.3, and the intensity-weighted size distribution shifts toward larger values but remains monomodal (See Supplementary Materials, Figure S1). It cannot be excluded that occasional aggregation of polyion-decorated vesicles may occur due to the non-uniform distribution of polyion, which is added at a very low concentration. However, it is undoubtedly that Chit modifies the aspect of the vesicle surface, which loses its transparency and appears fragmented in differently stained regions, which could be associated with patches of adsorbed polymer. Imaging of 𝜀-PLL-decorated liposomes in the absence of mucin (Figure 1, panel C) revealed vesicles with almost the same size as RIF-loaded liposomes but with a different surface structure, less transparent as in Chit-decorated liposomes due to the presence of polymer.

Panels D–E–F of Figure 1 show the samples after interaction with mucin. As expected from DLS, an increase of vesicle size is also observed by TEM as a consequence of the interaction with mucin. The increase is small for bare liposomes (Figure 1 panel D) while it is larger for Chito- and ε-PLL-decorated samples (Figure 1, panels E, F, respectively) and it could be connected to the favorable mucin-polycation interaction. It is immediately apparent that mucin does not induce vesicle rupture or restructuring and close-together roundish vesicles with net borders can be easily identified in polymer-coated samples.

**Figure 2:**
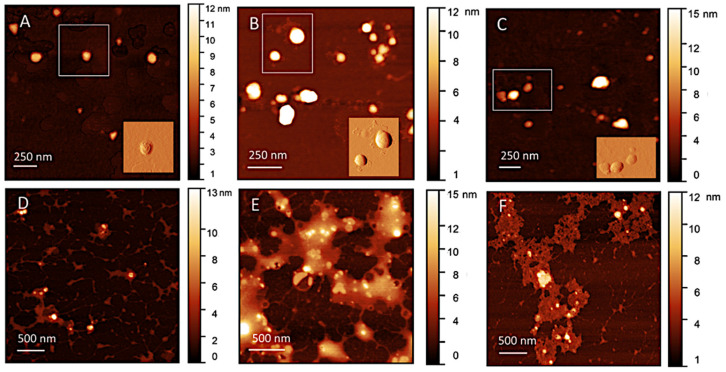
AFM topographical images (Height sensor channel) of liposomal formulations: LipoRIF (**A**), LipoRIF-Chito (**B**), LipoRIF-ε-PLL (**C**) observed as prepared and after interaction with mucin (**D**,**E**,**F**, respectively). The false color scale of height is shown on the left of each image. Insets in upper panels show the Prewitt-filtered images of the marked region.

AFM topographical images of RIF-liposomes and Chit/ε-PLL decorated RIF-liposomes, before and after interaction with mucin, are shown in Figure 2, panels A,B,C and D,E,F, respectively. Liposomes and polymer-decorated liposomes appear as large flattened spots with an average height in the range of 10–30 nm, due to the adsorption and drying on the hydrophilic mica. In polymer-decorated sample coexistence of single vesicles and small clusters is observed, as visible in TEM images. Vesicle opening and rupture are not observed [53], probably because the interaction with the support does not overcome the bending rigidity of the bilayer. As reported in several AFM investigations [54,55], liposomes can adsorb, flatten to a pancake-like structure and also undergo rupture to end up as single-bilayer disks by spreading in dependence of several factors as vesicle composition and properties of the support. Both liposomes and polymer-decorated liposomes result sufficiently flexible to flatten but their bilayer rigidity contributes to preserve their globular shape, as expected on the basis of anisotropy data.

RIF-liposomes can be easily recognized as well-separated spherical objects, with their roundish shape and regular contours (panel A), as it can be better observed in the inset reporting a vision with enhanced contrast obtained by application of horizontal Prewitt filter. AFM images reveal that in Chit-decorated liposomes (Figure 2, panels B) bumps and large irregularities appear on the contours of liposomes, as visible in the inset, and they are probably connected to the spreading of polymer and subsequent adhesion of the mica. Conversely, in ε-PLL-decorated liposomes the borders do not show any irregularities and they appear as regular globules (Figure 2, panels C). These differences can be connected to the different configurations assumed by the adsorbed polymers on the liposome surface [56], which have different chemical and structural properties, such as charge density and length.

AFM images give further details on what occurs after interaction with mucin, where the situation appears complex but interesting (Figure 2, panels D–F). Adsorbed mucin on LipoRIF is detectable as a small halo surrounding individual liposomes, or a few of them, while for polymer-decorated liposomes this halo considerably extends and connects several liposomes, which remain entrapped inside it. This confirms the different interaction of liposomes and polymer-decorated liposomes with mucin, in agreement to what is determined by absorbance studies.

### 3.4. Stability Studies

**Figure 3:**
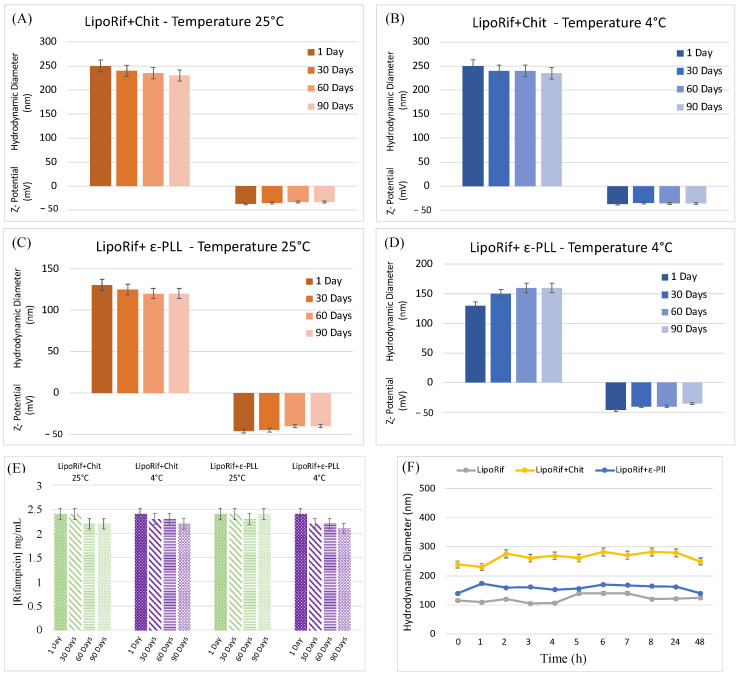
(**A**–**D**) Result of investigation on physicochemical stability of decorated liposomes until up to 90 days at 4 °C and room temperature. (**E**) Stability studies over time of Rifampicin-loaded liposomes at 2 different storage temperatures over a 90-day period. (**F**) Effect of culture media at different incubation times on hydrodynamic diameter and **ζ**-potential of liposomes.

To investigate the stability over time of polymer-decorated liposomal samples, the samples were stored at room temperature and 4 °C (temperature that could limit degradation lipids phenomena) for 90 days (Figure 3, panel A–D). It is possible to observe that no significant variations of hydrodynamic diameter or **ζ**-potential are detected over 90 days. Both Chit and ε-PLL-coated samples are stable at room temperature and 4 °C thanks to the high negative **ζ**-potential values which prevent aggregation or precipitation phenomena. In fact, as described in the final part of the introduction, the polyelectrolyte concentration used for vesicle coating has been chosen in order to have the proper amount of polymer to confer mucoadhesive property to the liposomes but not too high to reduce the **ζ**-potential and cause the aggregation of the polymer-decorated liposomes [27,40]. On the same samples, the amount of drug present was evaluated by UV analysis, to get information about RIF stability and exclude drug decomposition or degradation phenomena. As for measurements of size and **ζ**-potential on time, UV spectra were recorded immediately after LipoRIF + Chit/ε-PLL preparation and after 30, 60 and 90 days at room temperature and 4 °C. RIF concentration values were reported in Figure 3E,F. It is evident that drug amount remains constant during all the investigated time span. It can be concluded that the polyelectrolyte decoration does not influence the stability of the entrapped drug, thus confirming that the presence of polymer only affects the liposome surface, as above supposed.

### 3.5. Release Studies

**Figure 4:**
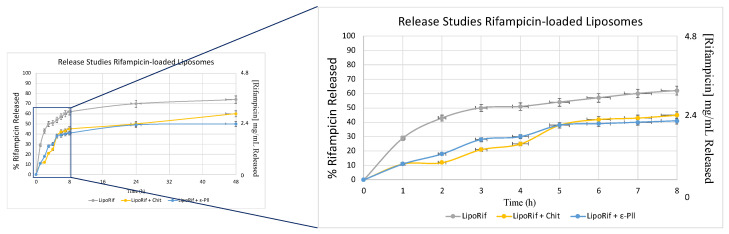
Rifampicin release profile until up to 48 h. Data were obtained as the mean of three independent experiments.

**Figure A1:**
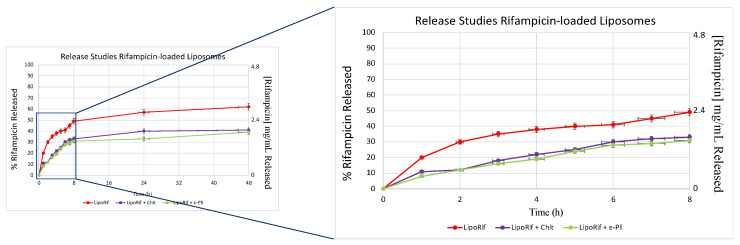
Rifampicin release profile until up to 48 h from liposomes post aerosolization. Data were obtained as the mean of three independent experiments.

**Figure A2:**
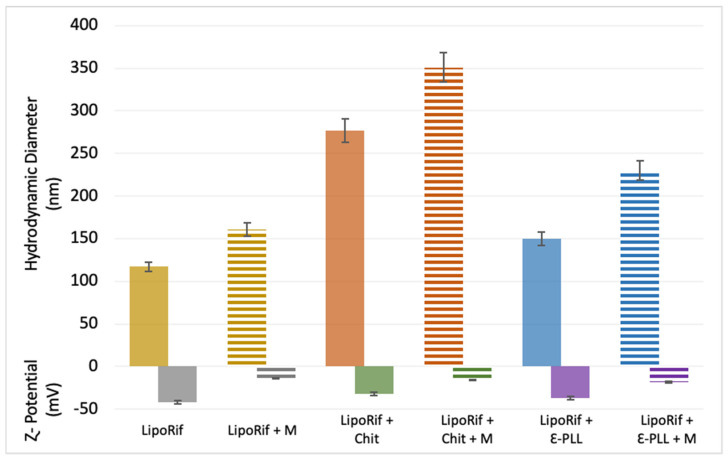
Hydrodynamic diameter (DH), and ζ-potential of decorated and undecorated liposomes in presence of mucin.

Figure 4 shows the amount of Rifampicin released from LipoRIF + Chit and LipoRIF + ε-PLL. The amount of RIF released from bare liposomes was significantly higher when compared to that of decorated vesicles. It is reasonable to think that the polymer decoration acts as an additional diffusion barrier which reduces the drug release rate [45]. It can be observed that all release curves (polymer-decorated and bare liposomes) display a biphasic trend, showing an initial burst release during the first 8 h followed by a gradual, slow release that lasts from 48 h. This behavior could be explained by the initial high concentration gradient across the membrane pores. Moreover, it is possible to observe a high RIF amount released by bare liposomes with respect to polymer-decorated ones probably due to the effect of the polymer [57]. Last, the release of drug could be affected by drug–polyelectrolyte interaction which could occur at this specific pH. Spectrophotometric measurements indicated that RIF is strongly absorbed by Chit at pH less than the pKa [58]. The same release profile has been obtained post nebulization experiment. This result confirms that aerosolized process doesn’t affect the release capability (Figure A1 Appendix A).

### 3.6. Biological Evaluation

**Figure 5:**
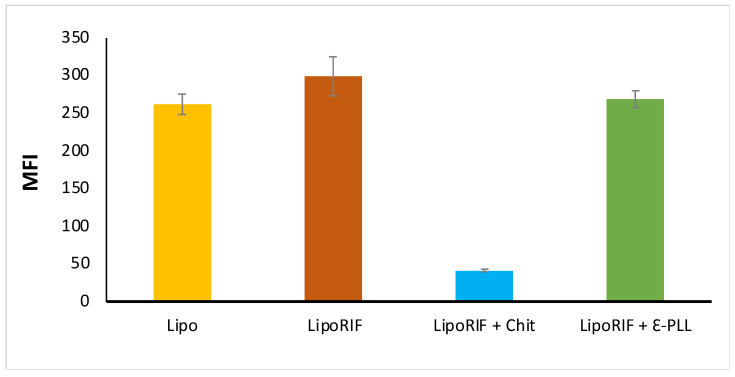
Liposome internalization analysis within macrophages. dTHP-1 (5 × 10^5^/mL) were stimulated for 18 h with empty liposomes (Lipo), drug-loaded liposomes (LipoRIF) or with polymer-decorated RIF-loaded liposomes (LipoRIF+ Chit LipoRIF+ ε-PLL), all containing calcein. Cells were collected and liposome uptake was analyzed by flow cytometry. Results of Mean Fluorescence Intensity (MFI) are shown as mean ± SD values obtained from two independent experiments.

To investigate how the presence of polymer could affect the efficacy of liposomes on the intracellular Mabs killing, the liposome uptake by dTHP-1 cells was evaluated. For this experiment, macrophages were stimulated for 18 h with fluorescent RIF-loaded liposomes coated or not with polymers. Figure 5 shows that Chit-decorated liposomes are internalized in dTHP-1 cells significantly less (≈7 times less) than LipoRIF and ε-PLL-decorated liposomes. Consistent with our observation, it has already been reported that Chit polymeric nanoparticles are poorly internalized in macrophages, where a dependence on nanoparticle surface chemistry in the cellular uptake and internalization pathways has been hypothesized [59]. No difference in uptake between LipoRIF and ε-PLL-decorated liposomes has been observed. It is reasonable to hypothesize that the different surface structure of Chit- and ε-PLL-decorated liposomes could be a factor affecting the cellular uptake and it will be the subject of future investigations focused on this specific aspect.

**Figure 6:**
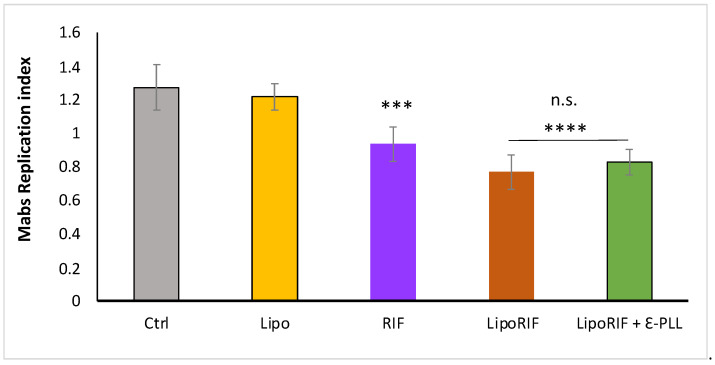
Polymer-decorated liposomes maintain their antimicrobial effect. To evaluate intracellular bacterial growth, dTHP-1 were infected with Mabs for 3 h at 37 °C at a MOI of 10. After infection, extracellular bacilli were eliminated by 1 h incubation with 250 ug/mL amikacin. Subsequently, dTHP-1 cells were treated for 18 h with unloaded liposomes, free RIF, RIF-loaded liposomes, RIF-loaded Ɛ-PLL-decorated liposomes. Finally, macrophages were lysed with deoxycholate 1% and then samples were diluted in PBS-tween 80 and CFU quantified by plating bacilli in triplicate on 7H10. Replication index was calculated as the ratio between the CFU obtained 18 h of treatment and those obtained immediately after infection, before the addition of the stimuli. The results are shown as mean ± standard deviation of the values obtained from triplicate of each condition and are representative of three different independent experiments. n.s. = non-significant, *** *p* < 0.001 and **** *p*< 0.0001 by two-tailed Student’s *t*-test.

**Figure A3:**
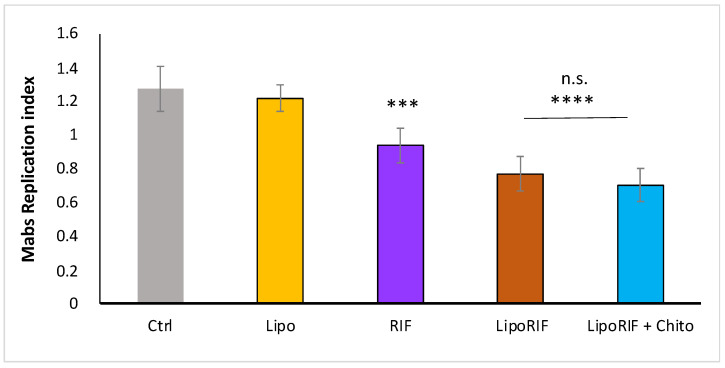
Antimicrobial effect of Chit-decorated RIF-liposomes. To evaluate intracellular bacterial growth, dTHP-1 were infected with Mabs for 3 h at 37 °C at a MOI of 10. After infection, extracellular bacilli were eliminated by 1 h incubation with 250 ug/mL amikacin. Subsequently, dTHP-1 cells were treated for 18 h with unloaded liposomes, free RIF, RIF-loaded liposomes, RIF-loaded Chit-decorated liposomes. Finally, macrophages were lysed with deoxycholate 1% and then samples were diluted in PBS-tween 80 and CFU quantified by plating bacilli in triplicate on 7H10. Replication index was calculated as the ratio between the CFU obtained 18 h of treatment and those obtained immediately after infection, before the addition of the stimuli. The results are shown as mean ± standard deviation of the values obtained from triplicate of each condition and are representative of three different independent experiments. n.s. = non-significant, *** *p* < 0.001 and **** *p* < 0.0001 by two-tailed Student’s *t*-test.

On these grounds, it was evaluated the biological effect of polymer-decorated liposomes with respect to empty liposomes and bare liposomes (LipoRIF). Here, it was focused on the effect of ε-PLL- decorated liposomes since the different cellular uptake of the two polymer-decorated liposome formulations makes the direct comparison on the Mabs intracellular killing not immediate. Results on ε-PLL-decorated liposomes are shown in Figure 6. It can be observed that ε-PLL-decorated liposomes are able to enhance Mabs killing in dTHP-1 cells in a similar fashion of undecorated LipoRIF. Interestingly, it is possible to conclude that the effectiveness of Rifampicin entrapped in Chit or ε-PLL-decorated liposomes is preserved, thus excluding polymer-drug interaction or degradation phenomena. Results on LipoRIF + Chit are shown in Figure A3 (Appendix B); an antimicrobial effect similar to the one observed for LipoRIF and ε-PLL- decorated liposomes is observed, despite their low internalization, which deserves a specific investigation to ascertain and understand.

Interestingly, ε-PLL does not interfere with both internalization and antimicrobial effects of Rifampicin-loaded liposomes in macrophages. It can be affirmed that ε-PLL- decorated liposomes entrapping Rifampicin may be suggested as valuable candidates for further in vivo validation studies to improve pulmonary antibiotic bioavailability and the therapeutic management of patients with Mabs infection.

## 4. Conclusions

The therapeutic management of pulmonary infections related to *Mycobacterium abscessus* (Mabs) presents many drawbacks connected to the intracellular localization of this pathogen, its wide drug-resistance, the side effects and the patient’s noncompliance associated with the long conventional treatments. In a recent investigation, it was shown that the encapsulation of Rifampicin (RIF) in anionic HSPC-DPPG liposomes can represent a promising strategy for the treatment of Mabs pulmonary infections thanks to the increased intramacrophage drug bioavailability connected with the nanocarrier encapsulation [29]. On this basis, this work has explored the possibility to coat RIF-loaded liposomes to increase the potential of in vivo benefits administering aerosolized mucoadhesive liposomes. In particular, coated liposomes could be able to reach the lung site infection and to interact with lung mucus overproduced in mucus-related disorders.

This work demonstrates that both chitosan and ε-poly-L-lysine confer, in vitro, mucoadhesive properties to HSPC-DPPG liposomes without compromising their stability and the capability to efficient entrap and release Rifampicin, which also remain unchanged after nebulization. Polymer-decorated RIF-liposomes show a very high drug entrapment efficiency and an efficient RIF release. It has to be pointed out that ε-PLL- decorated liposomes are internalized to a larger extent than chitosan-decorated liposomes by infected macrophages, where they carry out effective antimicrobial activity. To the best of our knowledge, this is the first evidence that ε-poly-L-lysine can be used as effective mucoadhesive and antimicrobial agent simultaneously.

All these findings support the evidence that ε-poly-L-lysine- decorated HSPC–DPPG liposomes can represent a valuable strategy for pulmonary delivery of Rifampicin by nebulization with several potential advantages in the treatment of the deep lung of infected patients.

## Figures and Tables

**Table 1:** Composition of the investigated samples: RIF-loaded liposomes (LipoRIF) and polymer decorated liposomes.

Sample	DPPG mg/mL	HSPC mg/mL	RIF mg/mL	Chit mg/mL	εPLL mg/mL	pH
LipoRIF	5	5	5	-	-	7.40 ± 0.01
LipoRIF + Chit	2.5	2.5	2.5	0.037	-	5.90 ± 0.01
LipoRIF + ε-PLL	2.5	2.5	2.5	-	0.05	6.10 ± 0.01

Errors are within 5%.

**Table 2:** Physicochemical features of polyion-decorated liposomal formulations, as prepared (before aerosolization). Data of bare liposomes are reported as a reference. Errors are the standard deviations (SD) of data.

Sample	D_H_ ± SD (nm)	PDI ± SD	ζ-pot ± SD (mV)	*E.E.* %	Anisotropy
LipoRIF	117 ± 2	0.22 ± 0.08	−2 ± 2	96 ± 2	0.34 ± 0.02
LipoRIF + Chit	277 ± 6	0.25 ± 0.04	−32 ± 2	95 ± 1	0.37 ± 0.01
LipoRIF + ε-PLL	150 ± 3	0.23 ± 0.07	−37 ± 1	95 ± 2	0.32 ± 0.01

**Table 3:** Characterization of liposomal formulations in the presence of mucin and evaluation of liposome–mucin interaction by determination of ΔA values.

Sample	D_H_± SD (nm)	PDI± SD	ζ-pot ± SD (mV)	∆A	∆A %
LipoRIF + M	161 ± 1	0.41 ± 0.02	−14 ±1	0.03 ± 0.01	8.0 ± 0.2
LipoRIF + Chit + M	361 ±3	0.41 ± 0.02	−16 ± 1	0.31 ± 0.01	77.0 ± 0.5
LipoRIF + ε-PLL + M	230 ± 3	0.36 ± 0.04	−18 ± 1	0.22 ± 0.01	55.0 ± 0.5
Mucin (M)	1623 ± 60	0.45 ± 0.08	−16 ± 1	-	-

## Data Availability

The data presented in this study are available on request from the corresponding author.

## Supplementary Materials

The following supporting information can be downloaded at: https://www.mdpi.com/article/10.3390/biom13060924/s1, Figure S1: Intensity-weighted size distribution of bare liposomes and polyion-decorated liposomal formulations obtained by NNLS analysis; Table S1: Hydrodynamic diameter of bare liposomes and polyion-decorated liposomal formulations obtained by NNLS analysis. Reference [60] is cited in the Supplementary Materials.

## Appendix A

### Appendix A.1. Post Nebulization Studies

Polymer-decorated liposomes, after nebulization by a jet nebulizer were analyzed in terms of size and size distribution, ζ-potential, entrapment efficiency, anisotropy and release capability and data obtained have been below reported (Table A1 and Figure A1). From the obtained data, it is possible to affirm that the nebulization process does not affect the physical chemical features of nebulized samples and their release capability.

**Table A1:** Physicochemical features of polymer decorated and bare liposomal formulations before and post aerosolization (pre and post, respectively).

Sample	D_H_ ± SD (nm)	PDI ± SD	ζ-pot ± SD (mV)	*E.E.* %	Anisotropy
LipoRIF pre	117 ± 2	0.22 ± 0.08	−42 ± 2	96± 2	0.30 ± 0.01
LipoRIF post	114 ± 1	0.29 ± 0.04	−47 ± 1	94 ± 1	0.33 ± 0.01
LipoRIF + Chit pre	277 ± 6	0.25 ± 0.04	−32 ± 2	95 ± 1	0.37 ± 0.01
LipoRIF + Chit post	283 ± 23	0.55 ± 0.01	−30 ± 2	92± 1	0.33 ± 0.01
LipoRIF + ε-PLL pre	150 ± 3	0.23 ± 0.07	−37 ± 1	95 ± 2	0.32 ± 0.01
LipoRIF + ε-PLL post	146 ± 2	0.29 ± 0.04	−40 ± 1	91 ± 1	0.36 ± 0.01

SD represents the standard deviation of data.

### Appendix A.2. Mucoadhesion Studies

In Figure A2, the values have been reported the values of hydrodynamic diameter (D_H_), and ζ-potential to better highlight, with the respect to Table 3, the size differences between decorated and undecorated liposomes also in presence of mucin.
