# Supplementary material for: Mucoadhesive Rifampicin-Liposomes for the Treatment of Pulmonary Infection by Mycobacterium abscessus: Chitosan or ε-Poly-L-Lysine Decoration
Source: Biomolecules. 2023 May 31;13(6):924. doi: 10.3390/biom13060924 (PMC10296137; doi:10.3390/biom13060924)
Supplement: Supplementary file 1 [file biomolecules-13-00924-s001.zip › biomolecules-2394002-supplementary.pdf]

Supplementary Information

Mucoadhesive Rifampicin-Liposomes for the Treatment of Pulmonary Infection by *Mycobacterium abscessus*: Chitosan or ε-poly-L-lysine Decoration

1. DLS: size distribution analysis

Dynamic Light Scattering (DLS) was used to determine the size distribution of the liposomal formulations by considering the intensity-weighted NNLS algorithm to analyze data [1.]

Figure S1. Intensity-weighted size distribution of bare liposomes and polyion-decorated liposomal formulations obtained by NNLS analysis.

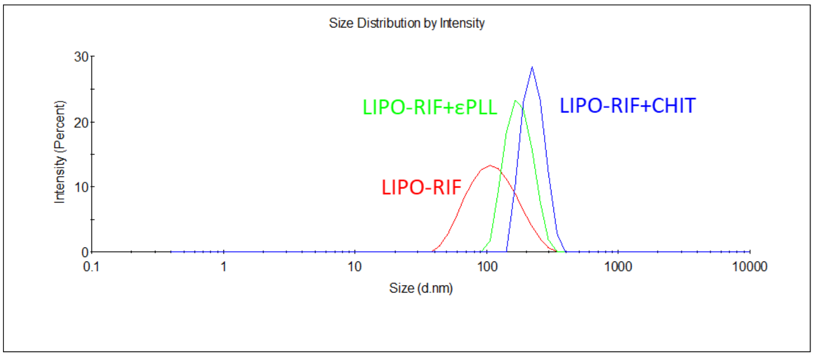

Table S1. Hydrodynamic diameter of bare liposomes and polyion-decorated liposomal formulations obtained by NNLS analysis. Errors are the standard deviations (SD) of data.

Sample	D_H± SD (nm)	width ± SD (nm)
LipoRIF	118 ± 4	49 ± 4
LipoRIF + Chit	228 ± 8	43 ± 2
LipoRIF + ε-PLL	179± 5	41 ± 3

1. Lawson, C. L., & Hanson, R. J. (1974). Solving Least Squares Problems, Prentice-Hall, Inc., Englewood Cliffs.
